# Supplementary material for: Hair-follicle associated pluripotent (HAP)-cell-sheet implantation enhanced wound healing in diabetic db/db mice
Source: PLoS One. 2024 Jun 14;19(6):e0304676. doi: 10.1371/journal.pone.0304676 (PMC11178214; doi:10.1371/journal.pone.0304676)
Supplement: Attachment — Submitted filename: Response to Reviewers.docx [file pone.0304676.s001.docx]

Journal Requirements:

When submitting your revision, we need you to address these additional requirements.

Please ensure that your manuscript meets PLOS ONE's style requirements, including those for file naming. The PLOS ONE style templates can be found at

https://journals.plos.org/plosone/s/file?id=wjVg/PLOSOne_formatting_sample_main_body.pdf and

https://journals.plos.org/plosone/s/file?id=ba62/PLOSOne_formatting_sample_title_authors_affiliations.pdf

We note that Figures 1 and 3 in your submission contain copyrighted images. All PLOS content is published under the Creative Commons Attribution License (CC BY 4.0), which means that the manuscript, images, and Supporting Information files will be freely available online, and any third party is permitted to access, download, copy, distribute, and use these materials in any way, even commercially, with proper attribution. For more information, see our copyright guidelines: http://journals.plos.org/plosone/s/licenses-and-copyright.

We require you to either (1) present written permission from the copyright holder to publish these figures specifically under the CC BY 4.0 license, or (2) remove the figures from your submission:

1. You may seek permission from the original copyright holder of Figure1 and 3 to publish the content specifically under the CC BY 4.0 license.

I created these figures using MS-PowerPoint and MS-paint, and the contents are my original data and photographs. I wonder which part is the copyrighted image, and who is the original copyright holder. I have no idea to resolve this problem now.

We recommend that you contact the original copyright holder with the Content Permission Form (http://journals.plos.org/plosone/s/file?id=7c09/content-permission-form.pdf) and the following text:

“I request permission for the open-access journal PLOS ONE to publish XXX under the Creative Commons Attribution License (CCAL) CC BY 4.0 (http://creativecommons.org/licenses/by/4.0/). Please be aware that this license allows unrestricted use and distribution, even commercially, by third parties. Please reply and provide explicit written permission to publish XXX under a CC BY license and complete the attached form.”

Please upload the completed Content Permission Form or other proof of granted permissions as an "Other" file with your submission.

In the figure caption of the copyrighted figure, please include the following text: “Reprinted from [ref] under a CC BY license, with permission from [name of publisher], original copyright [original copyright year].”

2. If you are unable to obtain permission from the original copyright holder to publish these figures under the CC BY 4.0 license or if the copyright holder’s requirements are incompatible with the CC BY 4.0 license, please either i) remove the figure or ii) supply a replacement figure that complies with the CC BY 4.0 license. Please check copyright information on all replacement figures and update the figure caption with source information. If applicable, please specify in the figure caption text when a figure is similar but not identical to the original image and is therefore for illustrative purposes only.

We suggest you thoroughly copyedit your manuscript for language usage, spelling, and grammar. If you do not know anyone who can help you do this, you may wish to consider employing a professional scientific editing service.

Whilst you may use any professional scientific editing service of your choice, PLOS has partnered with both American Journal Experts (AJE) and Editage to provide discounted services to PLOS authors. Both organizations have experience helping authors meet PLOS guidelines and can provide language editing, translation, manuscript formatting, and figure formatting to ensure your manuscript meets our submission guidelines. To take advantage of our partnership with AJE, visit the AJE website (http://learn.aje.com/plos/) for a 15% discount off AJE services. To take advantage of our partnership with Editage, visit the Editage website (www.editage.com) and enter referral code PLOSEDIT for a 15% discount off Editage services.If the PLOS editorial team finds any language issues in text that either AJE or Editage has edited, the service provider will re-edit the text for free.

I keenly realize the necessity to employ a professional scientific editing service. As I edited this manuscript until today to meet the deadline, the editing service could not be used. If you think the contents of this paper is acceptable to publish on your journal, I am going to copyedit the manuscript using such professional editing service.

Upon resubmission, please provide the following:

The name of the colleague or the details of the professional service that edited your manuscript

A copy of your manuscript showing your changes by either highlighting them or using track changes (uploaded as a *supporting information* file)

A clean copy of the edited manuscript (uploaded as the new *manuscript* file)

We noticed you have some minor occurrence of overlapping text with the following previous publication(s), which needs to be addressed:

https://www.jidonline.org/article/S0022-202X(23)02306-0/fulltext

In your revision ensure you cite all your sources (including your own works), and quote or rephrase any duplicated text outside the methods section. Further consideration is dependent on these concerns being addressed.

[Note: HTML markup is below. Please do not edit.]

Reviewers' comments:

Reviewer's Responses to Questions

Comments to the Author

1. Is the manuscript technically sound, and do the data support the conclusions?

The manuscript must describe a technically sound piece of scientific research with data that supports the conclusions. Experiments must have been conducted rigorously, with appropriate controls, replication, and sample sizes. The conclusions must be drawn appropriately based on the data presented.

Reviewer #1: Yes

Reviewer #2: Partly

2. Has the statistical analysis been performed appropriately and rigorously?

Reviewer #1: Yes

Reviewer #2: Yes

3. Have the authors made all data underlying the findings in their manuscript fully available?

The PLOS Data policy requires authors to make all data underlying the findings described in their manuscript fully available without restriction, with rare exception (please refer to the Data Availability Statement in the manuscript PDF file). The data should be provided as part of the manuscript or its supporting information, or deposited to a public repository. For example, in addition to summary statistics, the data points behind means, medians and variance measures should be available. If there are restrictions on publicly sharing data—e.g. participant privacy or use of data from a third party—those must be specified.

Reviewer #1: Yes

Reviewer #2: Yes

4. Is the manuscript presented in an intelligible fashion and written in standard English?

PLOS ONE does not copyedit accepted manuscripts, so the language in submitted articles must be clear, correct, and unambiguous. Any typographical or grammatical errors should be corrected at revision, so please note any specific errors here.

Reviewer #1: Yes

Reviewer #2: Yes

5. Review Comments to the Author

Please use the space provided to explain your answers to the questions above. You may also include additional comments for the author, including concerns about dual publication, research ethics, or publication ethics. (Please upload your review as an attachment if it exceeds 20,000 characters)

Reviewer #1: Major concerns

Authors constructed hair-follicle associated pluripotent (HAP)-cell-sheet for implantation to enhance wound healing in diabetic db/db mice. However, authors missed several critical factors.

1. The cell sheet contains a variety of cells, but which cell plays the critical role is unknown.

Precise role of each cell was not clear in the present study. We added discussion concerning the role of keratinocytes and macrophages.

2. There was no significant difference in histological results. Moreover, the epidermis and dermis in HAP group were thicker, which was not favorable for tissue regeneration.

I added Figure 4E to show the qualitative difference of healed epidermis and discussed about the ability of thicker epidermis and dermis.

3. Why could CD34-positive cells represent neovascularization, does the cell sheet still express CD34 after cell differentiation?

4.What is the biological role of CD34?

CD34-positive cells differentiate into multiple cell types as you pointed out, so I double stained with α-SMA to identify neovascularization (Figure 6A).

Minor concerns

1. The grammar needs to be improved thoroughly over the manuscript.

I realize the necessity to employ a professional scientific editing service. If this paper is acceptable to publish on this journal, I am going to copyedit the manuscript using professional editing service.

Reviewer #2: This study entitled 'Hair-follicle associated pluripotent (HAP)-cell-sheet implantation enhanced wound healing in diabetic db/db mice' focuses on utilizing HAP cells derived from hair follicles for treating excisional wound in diabetic conditions. The manuscript requires further refinement before it can satisfy the standards for publication.

major:

1. Concerning the use of flow cytometry for cell characterization from the HAP sheet:

a) There appears to be no use of isotype controls in the process.

Preliminary test using isotype control showed low background, so we didn’t use isotype controls in the process.

b) CD31 should be stained to exclude endothelial cells.

CD31 was added to Figure 2A. HAP-cells can differentiate into many types of cells including endothelial cells (lines 30, 62-65). We chose HAP-cell as donor cell for implantation because of this characteristic. So, we thought endothelial cells should not be excluded.

c) In Figure 2A, there are discrepancies in SSC-A (cell granularity) on the y-axis across the four charts. If these cells are from the same batch, uniformity is expected. An explanation for this variation is needed.

The figures are expressed as the result using three batches.

2. Regarding the transplantation of 5 × 10^5 cells for a 1 cm circular wound, I am skeptical about the effectiveness. Has there been any comparison with other cell types, such as keratinocytes? How many cells are typically implanted? Additionally, why is this particular type of cell, which requires a month to culture, considered more effective?

The total amount of transplanted cells were determined according to preliminary experiments. We selected this particular type of cell because it proliferates and differentiates into many types of cells, in expectation of multiple effects when clinically used.

3. The claim that the HAP-cell-sheet accelerates wound healing is not convincingly supported by the presented results: a) The sheet does not seem to properly align with the wound shape.

The cells are not fixed in the sheet, being able to proliferate and to migrate. Therefor I didn’t think to align the sheet properly with wound shape.

b) Re-epithelialization is crucial for wound closure. Has there been an assessment of when re-epithelialization occurs in db/db mice, particularly whether it happens sooner in the HAP-sheet treated group compared to the control group? It would be beneficial to perform Krt14 staining and H&E staining around the time of re-epithelialization.

I really appreciate your useful suggestion. I made additional experiments to find new findings (Figures 4 and 5).

c) The survival duration of the transplanted cells in relation to wound healing is uncertain. It's important to demonstrate the presence of transplanted cells throughout the healing process.

We confirmed the presence of transplanted cells at wound closure using LuminiCell Tracker 540- Cell Labeling Kit.

minor:

1. The structure of the article could be improved for better clarity. I suggest organizing it as follows: a) Isolation and characterization of HAP sheet cells. b) The role of HAP-cell-sheets in accelerating wound healing. c) The influence of HAP-cell-sheets on fibroblast growth and VEGF accumulation. d) The impact of HAP-cell-sheets on promoting angiogenesis. e) How HAP-cell-sheets may reduce inflammation.

I improved the manuscript according to your kind advice.

2. The process of isolating HAP is not sufficiently detailed: a) What methods are employed to prevent contamination?

All surgical procedures were done in a clean environment as described in reference 9.

b) Regarding lines 91-93, are the plates coated with Matrigel that detaches along with the cells? If Matrigel is part of the sheet, consider including a control group using only Matrigel sheets in both the wound scratch assay and in vivo healing studies. If there's no Matrigel, please clarify how the liquid cell pellet is maintained at the wound's center.

I described additional process on line 95. After culturing in ci-KIC iPS medium, cells were detached from the plates, washed, and cultured again in 10% FBS DMEM without Matrigel. Therefore, HAP cell sheets didn’t contain Matrigel.

Some HAP cell sheets were collected into a sheet-like cell pellet (described in line 149). A sheet-like cell pellet in 100 mL PBS was placed on the surface of the wound and covered with semi-occlusive adhesive dressing (lines 150-154). The cells were not fixed and maintained at the wound’s center.

c) Over the course of the three-week culture period, how frequently was the medium changed?

The medium was changed twice a week.

3. In Figure 2A, it would be beneficial to include the gating strategy used in the flow cytometry analysis.

Unfortunately, I could not include the gating strategy in the figure.

4. Concerning line 251, the statement "CD34-positive cells were observed around the follicles (Fig 2B)" needs clarification. What is the intended message here?

I added discussion concerning this point (lines 357-371).

5. In Figure 7A, please specify which section of the wound is being shown.

I added the specified section in Figure 6 (Figure 7 in the original manuscript).

6. PLOS authors have the option to publish the peer review history of their article (what does this mean?). If published, this will include your full peer review and any attached files.

If you choose “no”, your identity will remain anonymous but your review may still be made public.

Do you want your identity to be public for this peer review? For information about this choice, including consent withdrawal, please see our Privacy Policy.

Reviewer #1: No

Reviewer #2: No

[NOTE: If reviewer comments were submitted as an attachment file, they will be attached to this email and accessible via the submission site. Please log into your account, locate the manuscript record, and check for the action link "View Attachments". If this link does not appear, there are no attachment files.]

While revising your submission, please upload your figure files to the Preflight Analysis and Conversion Engine (PACE) digital diagnostic tool, https://pacev2.apexcovantage.com/. PACE helps ensure that figures meet PLOS requirements. To use PACE, you must first register as a user. Registration is free. Then, login and navigate to the UPLOAD tab, where you will find detailed instructions on how to use the tool. If you encounter any issues or have any questions when using PACE, please email PLOS at figures@plos.org. Please note that Supporting Information files do not need this step.
